# Properties of Composites Based on Recycled Polypropylene and Silico-Aluminous Industrial Waste

**DOI:** 10.3390/polym15112545

**Published:** 2023-05-31

**Authors:** George-Mihail Teodorescu, Zina Vuluga, Florin Oancea, Andreea Ionita, Jenica Paceagiu, Marius Ghiurea, Cristian-Andi Nicolae, Augusta Raluca Gabor, Valentin Raditoiu

**Affiliations:** 1 National Institute for Research & Development in Chemistry and Petrochemistry-ICECHIM, 202 Spl. Independentei, 060021 Bucharest, Romania; george.teodorescu@icechim.ro (G.-M.T.); florin.oancea@icechim.ro (F.O.); andreea.afilipoaei@icechim.ro (A.I.); marius.ghiurea@icechim.ro (M.G.); cristian.nicolae@icechim.ro (C.-A.N.); raluca.gabor@icechim.ro (A.R.G.); vraditoiu@icechim.ro (V.R.); 2 CEPROCIM S.A., 6 Preciziei, 062203 Bucharest, Romania; jenica.paceagiu@ceprocim.ro

**Keywords:** polypropylene recycled, fly ash, aluminosilicate waste, face masks, mechanical properties

## Abstract

There is an ever-growing interest in recovering and recycling waste materials due to their hazardous nature to the environment and human health. Recently, especially since the beginning of the COVID-19 pandemic, disposable medical face masks have been a major source of pollution, hence the rise in studies being conducted on how to recover and recycle this waste. At the same time, fly ash, an aluminosilicate waste, is being repurposed in various studies. The general approach to recycling these materials is to process and transform them into novel composites with potential applications in various industries. This work aims to investigate the properties of composites based on silico-aluminous industrial waste (ashes) and recycled polypropylene from disposable medical face masks and to create usefulness for these materials. Polypropylene/ash composites were prepared through melt processing methods, and samples were analyzed to get a general overview of the properties of these composites. Results showed that the polypropylene recycled from face masks used together with silico-aluminous ash can be processed through industrial melt processing methods and that the addition of only 5 wt% ash with a particle size of less than 90 µm, increases the thermal stability and the stiffness of the polypropylene matrix while maintaining its mechanical strength. Further investigations are needed to find specific applications in some industrial fields.

## 1. Introduction

It is well known that in recent decades, world governments have shown more interest in the capability of recycling polymeric composite materials due to the dangerous increase of waste and the pollution it causes to both nature and human health. Surgical face masks, being the most widely utilized and discarded personal protective equipment, represent such waste material, especially in recent years with the onset of the COVID-19 pandemic. Thus, researchers agree that it is necessary to develop innovative ways to recycle face masks waste to reduce their impact on the environment [1]. For example, Torres and De la Torre [2] and Sangkham [3] estimated the generation of face masks waste in locations such as Peru and Asia during the COVID-19 pandemic and proposed guides to better manage waste disposal and recycling of face masks to prevent further pollution and to control infection sources. As such, a study Carried out by Battegazzore et al. [4] followed to investigate the possibility and strategies of mechanically recycling surgical face masks in order to explore the recycled material’s potential in an industry. Other researchers have demonstrated the possibility of mechanical recycling and the use of waste surgical masks in construction as sound-absorbing materials [5] or for pavement base/subbase [6]. At present, there are only a few studies that seek to recover face mask waste and to repurpose it into new materials and even fewer that promote melt processing as a way to recycle the said masks. The review produced by Pourebrahimi [7] shows that it is possible to recycle and repurpose face masks into engineering materials, while Crespo et al. [8] have shown that the material obtained through mechanical recycling (extrusion–injection) of FFP2 respirator masks have better properties than polypropylene (PP) recycled from surgical masks [4] due to the blend of three thermoplastic polymers present in FFP2 face masks (PP, polyethylene and polyethylene terephthalate). This gives us the opportunity to explore possible ways of processing face masks as new polymer composites through melt processing methods. The preparation of polymer blends of such recycled materials is potentially an attractive way to reuse mixed waste streams at a lower cost. However, most polymers suffer from incompatibility, leading to poor phase morphology and mechanical properties, and as such, require compatibilizers to improve the stability of the blends. Varghese et al. [9] have demonstrated that maleic anhydride can act as a bridge between polypropylene recycled from used N95 medical mask filters and acrylonitrile butadiene rubber. Recycled polypropylene from recovered face masks can be easily mixed with different types of natural and synthetic (nano)fillers. Usually, polypropylene is processed by conventional technologies, such as extrusion and injection molding to make parts for various industries, thus, having great potential in the manufacturing of composites and nanocomposites [10]. Since certain types of surgical face masks have a composition of 100% polypropylene in their fabric, they present potential to be processed in the same way.

Interest in the use of fly ash, a waste material resulting from the general process of coal combustion, has increased in the last 10 years due to its silico-aluminous composition. Fly ash forms at temperatures in the range of 920–1200 °C and is collected as precipitator ash (solid particles) and cenospheres (hollow microspheres) that float on collection ponds. Disposing of this waste requires large quantities of land, water, and energy and also, due to its fine particles, if not managed well, it can become airborne and present a major health hazard. Therefore, many studies have been performed in order to repurpose this waste, although the general approach was to introduce fly ash as a low-cost filler in cement, individually or in combination with natural/synthetic fibers [11,12] or epoxy [13,14,15] compositions in the construction industry, or as a reinforcing agent in polymer or metal compositions [16]. There are a few advantages to the introduction of fly ash in a polymer composite. Raja et al. [17] discovered that the presence of 10 wt% of fly ash in unsaturated polyester composite with 20 wt% of glass fiber can lead to an increase in mechanical properties due to good interaction between the filler and the polymer matrix, resulting in a better stress transfer and lower chance of crack formation. Some attempts have been made to incorporate several concentrations of fly ash with different particle sizes into a PP matrix. It has been demonstrated that ash with small particle sizes leads to obtaining polymer composites with much better properties compared to ash with large particle sizes. Nath et al. [18] demonstrated that, compared to PP, injection-molded PP composites with 20–60% fly ash with a particle size of 5–60 µm exhibited a 10–60% higher modulus of elasticity, but a linear decrease in tensile strength at 25 °C and a substantial increase in tensile and impact strength at 50–70 °C. Composites based on PP with 10% ash with 53–75 µm particle sizes showed an improvement in flexural strength by approx. 30% [19].

To achieve a homogeneous compPP osite, dispersion and compatibility agents are usually required, especially in the case of materials with weak polar reactive groups. Due to its poor miscibility with clay silicates and hydrophobic character, the polypropylene in face masks requires the presence of a dispersion/coupling agent in order to increase the compatibility between the polymer matrix and the filler. Such an example was explored by Chrissopoulou and Anastasiadis [20], who studied functional compatibilizers between polyolefin and layered silicate materials and discovered that, generally, commercial macromolecular compatibilizers (polyolefin modified with maleic anhydride) are commonly used in order to improve the interfacial bonding between the polymers and the fillers. Joseph et al. [21] have reported an improvement of tensile and flexural strength by up to 75% and 170%, respectively, as well as in the impact strength by up to 270% for composites based on recycled polyethylene terephthalate (PET) and 5–15% fly ash treated with 1% 3-aminopropyltrimethoxysilane/vinyltrimethoxysilane coupling agents. It should also be noted that the spherical shape of fly ash particles improves the distribution rate of the filler in the polymer matrix, as studied by Kutchko and Kim [22]. For a better dispersion of fillers in the polymer matrix, agents such as poly(propylene glycol adipate) can be used. The studies performed by Hao et al. [23] and Zhao et al. [24] showed that poly(propylene glycol adipate) could improve the miscibility between the composite components since it acts as a plasticizer and also improves the general mechanical properties of composites.

The objective of this work is to study the properties of composites based on recycled polypropylene recovered from face masks for medical use and three types of aluminosilicate industrial waste in order to determine potential applications for these waste materials. The utilization of ash waste and face mask waste can create value for these materials and help the environment. Incorporation of ash into the recycled polypropylene matrix will be advantageous both for industry, for potential uses in the automotive or construction industry, where increased polypropylene stiffness is required without compromising mechanical strength, for obtaining performance parts from thermoplastics, and for the waste management commission for reducing the polluting effect of waste on the environment by reducing its quantity and its rational valorization through reconversion into an advanced polymer composite with prospects for industry. From a social point of view, the utilization and repurposing of waste materials may lead to an increase in recycling behavior for both regular consumers and industrial manufacturers. From an industrial perspective, utilizing ash waste and face mask waste may lead to the replacement of already used commercial polymers or reinforcing fillers since nowadays there is an increasing interest in developing products made from recycled/recovered sources while maintaining good general mechanical and thermal properties, which is an essential issue to building a circular economy.

## 2. Materials and Methods

### 2.1. Materials

Worn single-use face masks in regulation with STANDARD SR EN 14683+AC: 2019, with 3 layers and a 100% polypropylene composition were used. Three aluminosilicate ashes samples were used as reinforcing fillers, of which two were from the coal thermal power plant of Govora (CET Govora S.A., Râmnicu Vâlcea, România), marked with CG (fly ash from an electro filter) and GHG (a mixture of bottom and fly ash hydraulically discharged into the landfill (the humidity of sample was 32.8%), respectively, and one resulting from the process of obtaining basalt wool, marked with CVB (from an electro filter). The dispersion agent, poly(propylene glycol adipate) (P) (Solventul S.A., Timisoara, Romania), a clear yellow-brown liquid with a density at 20 °C = 1.150–1.200 g/cm^3^; viscosity at 20 °C: 11,000–16,000 cP; acidity: max. 2.0 KOH/g; saponification index: 550–585 mg KOH/g; and a compatibilizing agent, polypropylene-graft-maleic anhydride (PP-MA) (Polybond 3200, Crompton, Middlebury, CT, USA) with a density = 0.91 g/cm^3^ and a melting point of 157 °C were used.

### 2.2. Preparation of Composites

The single-use face masks are formed of 3 components: face mask, ear loops, and nose wire. These components were manually separated from each other using scissors and the face masks were recovered, disinfected on both faces with sanitizing alcohol, dried for 2 h at 80 °C, and thermomechanically recycled to obtain recycled polypropylene (PP), which was further used as a polymer matrix. The ash samples were dried to a moisture content of <0.1%, after which they were ground to obtain fine and very fine powders (particles with sizes < 125 µm for the CG sample and particles < 90 µm in the case of CHG and CVB samples, respectively). The oxidic chemical composition, determined according to SR EN 196-2 and the physical characteristics of the 3 ashes, are presented in Table 1.

Prior to the processing of the samples, the surface of fly ash was treated with a 30 wt% dispersion agent P in order to obtain a better homogeneity of the composites. The treated aluminosilicate ashes were denoted as CGP, CHGP, and CVBP. The modification was carried out in dynamical conditions, at 80 °C for 1 h. Granules of both PP and PP composites with 6.5 wt% treated aluminosilicate ash and 2.5 wt% PP–MA were obtained in dynamic conditions by melt processing, using a co-rotating twin screw extruder type Leistritz LSM 30.34 (Leistritz Extrusionstechnik GmbH, Nürnberg, Bayern, Germany), at an extrusion temperature of 170 ± 5 °C, screw rotation speed of 220 rot/min and feed rate of 100 rot/min (5 kg/h). The extruded filaments were cooled down and granulated with a drawer-Leistritz Granulator system (Leistritz Extrusionstechnik GmbH, Nürnberg, Bayern, Germany). For mechanical testing, PP and PP composite granules were injected using an Engel Victory VC 60/28 TECH (Engel, Schwertberg, Austria) injection-molding machine, into standard test specimens for tensile and impact tests. The temperature profile ranged from 180 to 200 °C with an injection speed of 2 mm/min, an injection pressure of 600 kg/cm^2^, a holding pressure of 350 kg/cm^2^, and a mold temperature of 50 °C.

### 2.3. Characterization

#### 2.3.1. Fourier Transform Infrared Spectroscopy Analysis (FTIR)

The chemical structure of ash before and after treatment with the dispersion agent P was investigated with a JASCO 6300 FT-IR spectrophotometer (JASCO Int. Co., Ltd., Tokyo, Japan) equipped with a Golden Gate ATR (crystal of diamond) from Specac Ltd. (London, UK). FTIR spectra were recorded in the range 4000–400 cm^−1^ with 30 scans per spectrum and a resolution of 4 cm^−1^. To better distinguish the crystalline forms of recycled polypropylene, as well as the changes in the crystalline structure of the PP in the composites, Fourier Transform Infrared (Jasco FTIR 6300, Tokyo, Japan) spectrophotometer equipped with a Pike Mid-IR IntegratIR integrating sphere (3-inch diameter, highly reflective gold-coated sphere), operating in the range 5000–500 cm^−1^ and with a wide-band MCT (liquid nitrogen) detector was used.

#### 2.3.2. Thermal Characterization

Thermogravimetric analysis (TGA) was performed on TA-Q5000IR (TA Instruments, New Castle, DE, USA) that used nitrogen as the purge gas (flow rate: 40 mL/min). Each thermogram was acquired between 25 and 700 °C with a heating rate of 10 °C/min. Duplicate samples weighed in a platinum pan (7–8 mg) were used for each measurement.

Differential scanning calorimetry (DSC) Q2000 equipment (TA Instruments, New Castle, DE, USA) was used to determine the melting and crystallization behavior of the samples. A single run consists of the heat–cool–heat (HCH) method, which began with an equilibration at −40 °C for 3 min. The material was brought to 240 °C, held for 2 min, then chilled to −40 °C, held again for 2 min, and reheated up to 240 °C with the cooling/heating rate of 10 °C/min. All measurements were carried out with a flow rate of 25 mL/min and under 5.0-grade helium. The melting temperature, Tm (maximum of the melting endotherm), the crystallization temperature, Tc (maximum of the crystallization exotherm), and the degree of crystallinity, Xc, calculated according to the method presented elsewhere [25], were analyzed.

#### 2.3.3. Dynamic Mechanical Analysis (DMA)

DMA Q800 (TA Instruments, New Castle, DE, USA) was used to measure the loss factor (tan δ), loss modulus (E″) and storage modulus (E′) of the samples as a function of temperature. Samples measuring 35 × 10 × 4 mm (length × width × thickness) were scanned across the temperature range of −40 to 140 °C with a heating rate of 3 °C/min, under a frequency of 1 Hz and at an amplitude of 20 µm.

#### 2.3.4. X-ray Diffraction Analysis (XRD)

X-ray diffraction measurements were carried out with a Rigaku Smartlab (Rigaku Corporation, Tokyo, Japan) diffractometer using a CuK_α1_ radiation (λ = 1.5406 Å). In this experiment, the accelerating voltage of the generator radiation was set at 45 kV and the emission current at 200 mA. The diffractograms were recorded at room temperature in parallel beam geometry over 2θ = 5° to 90°, with 0.02° intervals, continuously, at a scan rate of 4°/min. The interplanar distance (d) of PP composites was calculated from the Bragg equation:

(1) nλ=2dsin⁡θ

where n represents the reflexion order, λ is the wavelength of X-rays, θ is the diffraction angle, and d is the distance between the planes of the crystalline network which produces the diffraction. The crystallite sizes, FWHM—full width at half maximum intensity of the diffraction peak, height, and intensity values were obtained using the Rigaku Data Analysis Software PDXL 2, and the identification of crystalline phases was made using the Powder Diffraction File™ (PDF) of the International Centre for Diffraction Data (ICDD).

Individual diffraction maxima were extracted by a curve-fitting process from the diffraction profiles. A peak deconvolution program (PDXL: Integrated X-ray Powder Diffraction Software) was used, assuming a broad peak at approximately 2θ of 18° representing the amorphous phase. The crystallinity index was calculated by relating the area of the diffraction maxima of the crystalline phases to the total area, resulting from the elimination of the reference line.

#### 2.3.5. Mechanical Properties Analysis

Instron 3382 Universal Testing Machine (Instron Corporation, Norwood, MA, USA) of 10 kN load capacity was used to determine the tensile properties of the samples according to ISO 527. Seven specimens were used for each test, with 2 mm/min for the modulus of elasticity and 50 mm/min for the tensile strength. The impact strength of the samples was determined for seven specimens per test using a Zwick HIT5.5 Pendulum Impact Testers (Zwick Roell AG, Ulm, Germany), with a pendulum of 2.75 J and Izod notched impact method according to ISO 180/A.

#### 2.3.6. Nanomechanical Analysis

A TI Premier system (Hysitron Inc., Minneapolis, MN, USA) was used to perform the nanoindentation and nanoscratching tests using a three-side pyramidal Berkovich tip (total angles of 142.35 deg and radius of curvature of 150 nm). Nanoindentation tests were performed with a normal load of 10,000 µN, using the trapezoidal load function (5 s loading, 2 s hold, 5 s unloading) to determine the load-displacement curves and the values of hardness (H) and reduced modulus (Er), calculated using the Oliver–Pharr method. Nanoscratching was performed using a constant load scratch of 1000 µN only load function to record the lateral force and lateral displacement as a function of time in order to determine the coefficient of friction (µ = LF, µN/NF, µN). A scanning probe microscopy (SPM) mod of the TI Premier system was used to characterize the surface topography of the samples, and a representative 25 µm in situ SPM image was obtained before and after scratching. These images are necessary for the nanoscratch depth analysis (surface roughness and coefficient of friction) and post-test qualitative surface characterization.

#### 2.3.7. Scanning Electron Microscopy (SEM)

The morphological characteristics of the polypropylene/ash composites were analyzed by SEM using a Hitachi TM4000 plus microscope (Hitachi, Tokyo, Japan) with an accelerating voltage of 15 kV. Prior to the SEM analysis, the tensile test specimens were fractured in liquid nitrogen and sputter-coated with a thin layer (5 nm) of gold using a Q150R Plus (Quorum Technologies, SXE, Lewes, UK).

## 3. Results

### 3.1. Characterization of Modified Ashes Compositions

#### 3.1.1. SEM and EDX Analysis

**Figure 1:**
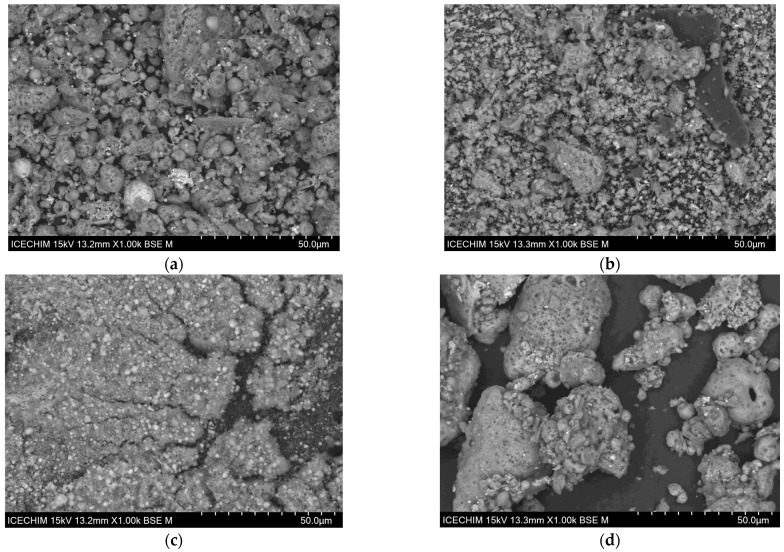
SEM surface morphology of (**a**) CG ash powder; (**b**) CHG ash powder; (**c**) CVB ash powder; (**d**) CGP mixture.

Figure 1 shows the different surface morphologies for each ash powder and one mixture of ash powder with the dispersion agent. The most noticeable difference between the ashes used is the irregular shape of the particles in each powder. Except for the CG powder in Figure 1a, where a few spherical particles can be seen, CHG and CVB powders have an irregular particle shape, as seen in Figure 1b,c. This finding is explained by the fact that the CG ash (fly ash from electro filter) was subjected to light grinding, partially preserving the initial shape of the particles (the spherical ones), while the initial structure of the other two ashes was destroyed by the very advanced grinding. Following the treatment of the fly ash surface with the dispersant, an agglomeration of the particles occurred—the particles being bound together in chunks that can crumble easily, as can be seen in Figure 1d. The same behavior also occurred for the CHGP and CVBP mixtures.

#### 3.1.2. X-ray Diffraction Analysis

**Figure 2:**
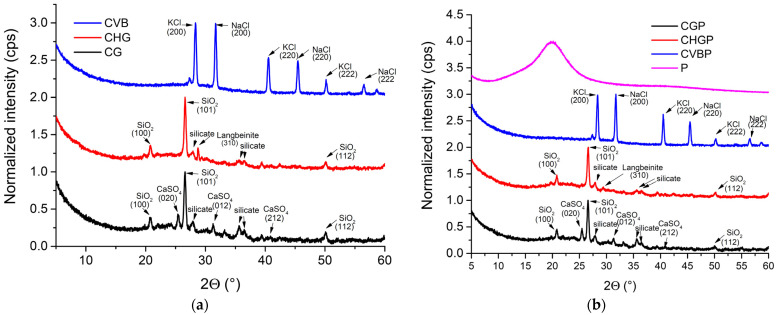
XRD patterns of (**a**) untreated ash powders CG, CHG, and CVB, and (**b**) treated ash powders CGP, CHGP, and CVBP.

The XRD patterns for the untreated and treated ash are shown in Figure 2.

In Figure 2a, in the range 2θ = 5° to 60°, we can see the characteristic peaks of the component crystalline phases of the three ashes. Thus, the characteristic peaks of some crystalline minerals composed of silica and silicates can be observed. In the CG ash, silica (quartz) at 2θ = 20.77°, 26.55°, 36.53°, and 50.16°; anorthite and/or albite (calcium aluminum silicate) at 2θ = 21.97°, 27.87°, and 33.13°; magnesioferrite (magnesium iron oxide) at 2θ = 35.67°; and anhydrite (calcium sulfate) at 2θ = 25.43°, 31.26°; and 40.83°, were detected. In the case of CHG ash, apart from the silica, anorthite and/or albite and magnesioferrite phases, the characteristic peak of another mineral, namely langbeinite (potassium magnesium sulfate) at 2θ = 28.76° can be observed. The constituent phases resulting from XRD are consistent with the oxide chemical composition results shown in Table 1. The XRD patterns for CVB ash, also in accordance with the oxide chemical composition results, were totally different from those for CG and CHG, respectively. We can see the characteristic peaks of two natural minerals, sylvine (potassium chloride) at 2θ = 28.23°, 40.51°, 50.17°, and 58.54° and halite (sodium chloride) at 2θ = 27.32°, 31.67°, 45.43°, and 56.4°. The characteristic peaks for crystalline silica do not appear in the case of this ash because almost all the silica in the composition is reactive, amorphous silica. Similar diffractograms were obtained for the treated ashes (Figure 2b). However, it was observed that, in general, there was a trend towards a decrease in peak height (more obvious for the strongest diffraction peaks, (101) diffraction peak for CGP and CHGP, and (200) diffraction peak for CVBP), and an increase in FWHM and crystallite size, which is evidence of ash-dispersant interactions. Thus, for the CVBP and CHGP samples, there was a decrease in height of 22% and 4%, respectively, and an increase in FWHM and crystallite size of 3% and 11%, respectively (Table 2 and Table 3). These results suggest a disorder in the mineral structure, greater in CHGP than in CVBP, with evidence of favorable interactions between ash and agent P. The exception is the CGP sample, where a slight tendency to increase the height of the peaks was found (by approx. 6.5%), while the rest of the dimensions remained approximately constant. In the case of this sample, the ordered structure was maintained, proving a weak interaction between the ash and the P agent.

#### 3.1.3. FTIR Analysis

**Figure 3:**
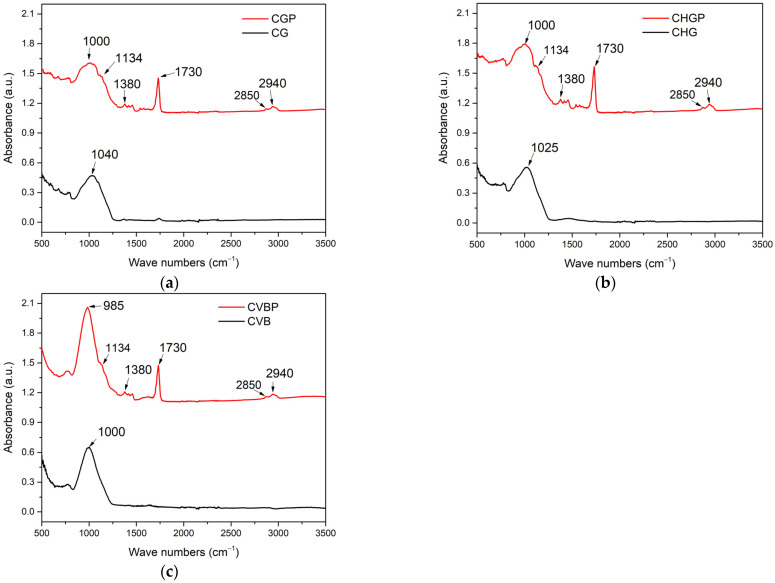
FTIR spectra for (**a**) CG and CGP; (**b**) CHG and CHGP; (**c**) CVB and CVBP.

The results of FTIR analysis of ashes before and after surface treatment with agent P are shown in Figure 3a–c.

There is general agreement in the research that all aluminosilicates generally exhibit a main broad and strong absorption peak at about 1080 cm^−1^ (depending on the composition) [26]. This band is usually a superposition of bands situated close to each other and related to the Si-O bond stretching vibration coupled with Si-O-Si and O-Si-O bending vibration motion in SiO_4_ tetrahedron. In each image, there is a strong absorption peak that ranges from 1000 cm^−1^ to 1040 cm^−1^, specific to the ash powders. With the addition of the dispersion agent P, several specific peaks appear, such as a strong absorption peak at 1730 cm^−1^ for the stretching of the C=O carbonyl group, while at 1380 cm^−1^, the CH bending peak is present, and at 1134 cm^−1^, the C-O-C stretching peak appears. Other noticeable peaks appear at 2940 cm^−1^ and 2850 cm^−1^, associated with the asymmetric and symmetric vibration of hydrogen in methyl groups. All these peaks confirm the presence of P in the ash powder, rendering the treatment of the ash a success.

#### 3.1.4. Thermogravimetric Analysis

**Figure 4:**
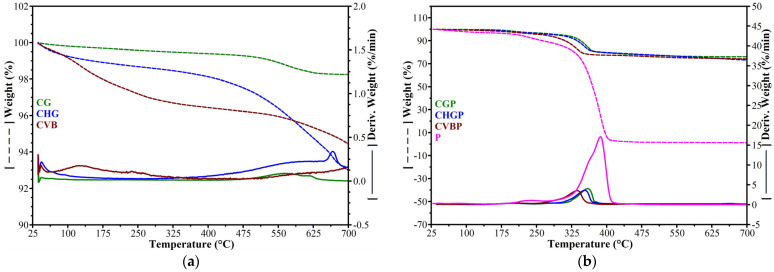
TGA curves for (**a**) untreated ash powders CG, CHG, and CVB; (**b**) treated ash powders CGP, CHGP, CVBP, and P.

TGA was performed on the dispersion agent P and the treated and untreated ash powder (Figure 4).

From Figure 4a, it can be seen that the untreated ash powders present significant differences from each other. CVB ash is the first one to begin the decomposition process, and up to 230 °C, it lost approx. 2.6%, compared to CHG and CG, which lost approx. 1.3% and 0.4%, respectively. Up to approx. 475 °C, the decomposition process continues slowly, with the samples being maintained in the same order of thermal stability: CG > CHG > CVB. Above 475 °C, the decomposition speed increases, with the highest percentage of losses being recorded for CHG ash, so that the residue at 700 °C was lower than in the case of CVB ash. A study performed by Sciubidło and Nowak [27] pointed out the existence of multiple local minima during the loss of weight in fly ash. The first loss of weight occurs at the local minimum of 50 °C, which is caused by water evaporation. The second loss of weight can be seen at 500 °C for the CVB ash associated with the dehydration of Ca(OH)_2_. In Figure 4b, there is an obvious difference between the treated ashes and P caused by their differences in organic and inorganic nature.

In the analyzed temperature range (25 °C–700 °C), the treated ashes show mainly four stages of decomposition—the same as agent P. In the first stage of decomposition, in the RT-100 °C range, the amount of water removed was generally lower after agent P treatment (0.60% and 0.63% for CHGP and CVBP vs. 0.75% and 0.83% for CHG and CVB). The exception was CGP ash, where the loss was higher after agent P treatment (0.26% vs. 0.19%). In this interval, agent P lost 2.17%. Up to 230 °C, CGP, CHGP, and CVBP-treated ashes lost 2.74%, 2.96%, and 3.63%, respectively, but in comparison with the untreated ashes, the weight loss varied in order: CGP > CHGP > CVBP. In this interval, agent P loses 6.63%. This order of weight loss is also preserved in the third stage of decomposition, in the temperature range 230 °C–295 °C. On the fourth stage of decomposition, in the temperature range 295 °C–410 °C, losses increased substantially (16.27%, 15.51%, and 15.19%) in the order: CGP > CHGP > CVBP. In this interval, agent P decomposed almost completely, with the maximum speed at a temperature of 386.6 °C. The theoretical value of the residue at 700 °C, for the three treated ashes, taking into account the weight loss of each untreated ash (98.25% for CGP, 94.44% for CVBP, and 92.99% for CHGP) and the agent P (1.24%), was 75.86% for CGP, 72.93% for CVBP, and 71.82% for CHGP. Compared to the theoretical values, the residue values at 700 °C, shown in Table 4, are higher (0.3–2% variation from the theoretical value), which could be evidence of good interactions between the ash and the agent P (possibly through the –OH groups), reflected in the increase in the thermal stability of the treated ash. The difference between the residual value presented in Table 4 and the theoretical value varies in order CHGP > CVBP > CGP and provides a measure of the interaction degree between the ash and agent P.

The TGA results are in good agreement with the XRD analysis results.

### 3.2. Characterization of PP Composites

#### 3.2.1. X-ray Diffraction Analysis

**Figure 5:**
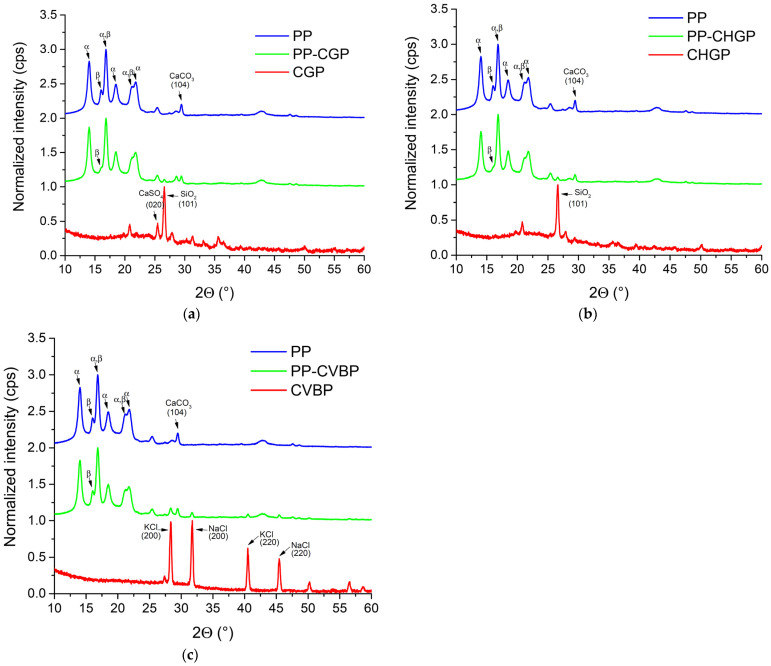
XRD patterns for the PP composites in comparison with PP and the corresponding treated ash (**a**) PP–CGP composite; (**b**) PP–CHGP composite; (**c**) PP–CVBP composite.

The XRD patterns for PP composites are presented in Figure 5. According to Foresta et al. [28], the peaks of the α and β crystalline phases of PP were assigned; the α crystalline phase was identified at 2θ = 14.02°, 16.82°, 18.49°, 21.77°, and 25.44° which correspond to the (110), (040), (130), (131), and (060) crystallographic planes. The β crystalline phase was identified at 2θ = 16.01°, which corresponds to the (300) crystallographic plane [29]. A peak characteristic for CaCO_3_ at 2θ = 29.42° can also be seen, which corresponds to the (104) crystallographic plane (PDF card No. 01-086-5297). The use of CaCO_3_ in the case of the PP blown films is known to improve both the productivity of blown extrusion and the mechanical properties of the blown film [30]. After the addition of treated ash, the characteristic peaks for PP were more or less detected. It was observed that with the addition of CGP and CHGP ashes, the characteristic peak for the β crystalline phase decreased greatly and appeared as a very small shoulder (Figure 5a,b). The same thing did not happen after the addition of CVBP ash when the peak corresponding to the β crystalline phase was clearly visible (Figure 5c). The presence of the β crystalline form in polypropylene and PP composites is very important because the content of the β form is responsible for the impact strength [31]. In the PP composites, the characteristic peaks for the α and β crystalline phases of PP have different and lower intensities compared to those in PP (Table 5).

After the deconvolution of the diffraction maxima, the relative amount of polypropylene β to polypropylene α ($K_{\beta }$) was calculated according to the Turner–Jones formula [29]:

(2) $$K_{\beta }=\frac{H_{\beta (300)}}{H_{\alpha (110)}+H_{\alpha (040)}+H_{\alpha (120)}+H_{\beta (300)}}\cdot 100\%$$

where $H_{\alpha (ikj)}$ represents the intensity of the respective diffraction maxima of polypropylene α and $H_{\beta (300)}$ the intensity of the diffraction maximum corresponding to the diffraction plane (300) of polypropylene β in counts per second (cps).

The value obtained for K_β_ decreases in the order PP > PP–CVBP > PP–CHGP > PP–CGP (Table 5). These results highlight the existence of an interaction between PP, PP–MA and treated ash, stronger in the case of PP–CVBP and PP–CHGP composites.

The crystallinity index determined from XRD measurements was 63.5 for PP–CVBP, 65 for PP–CHGP, 70 for PP–CGP, and 73.5 for PP. It is observed that the crystallinity indices vary in the same order as the intensity of the peaks corresponding to the α crystalline phase PP > PP–CHGP > PP–CGP > PP–CVBP. This behavior should also be reflected in the mechanical properties of the composites knowing that the α crystalline form leads to high strength and stiffness values [31]. The peaks with the highest intensity, characteristic of the ash used, can also be observed in the PP composites (Figure 5a–c).

#### 3.2.2. FTIR Analysis

**Figure 6:**
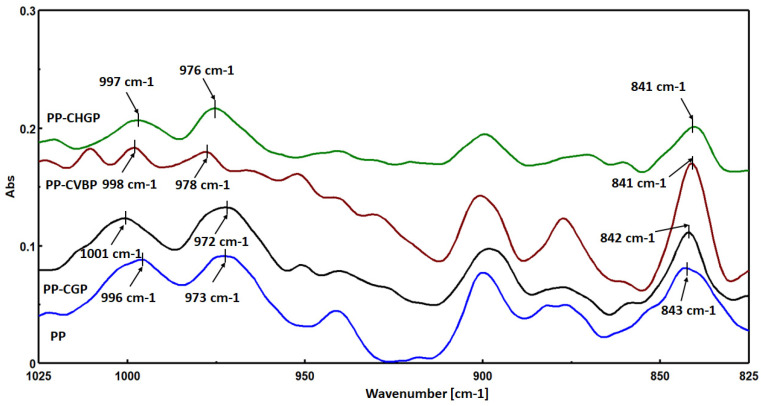
FTIR spectra of PP, PP–CGP, PP–CHGP, and PP–CVBP composites.

Figure 6 shows the FTIR spectra for the recycled PP and its composites containing 6.5% treated ash.

The peaks marked in Figure 6 in the range of 972 cm^−1^ to 1001 cm^−1^ are all associated with the -CH_3_ asymmetric rocking vibration and C-C asymmetric stretching vibration of PP, while the peaks marked in the range of 841 cm^−1^ to 843 cm^−1^ are associated with the -CH_2_ rocking vibration of PP. The peak at 996 cm^−1^ corresponds to the crystalline phase of PP (α and β forms), and the peak at 973 cm^−1^ corresponds to both the crystalline phase and the amorphous phase of PP [32]. The ratio between the maximum intensities of the two peaks can be used as a measure of the degree of crystallinity [32]. In our case, it was found that the degree of crystallinity of the composites varied when compared to neat PP. Thus, compared to PP, which had a degree of crystallinity of 0.894, the PP–CHGP composite showed the lowest degree of crystallinity (0.673), while the PP–CVBP composite showed the highest degree of crystallinity (1.029). The PP–CGP composite had crystallinity about 7% lower than that of PP (0.827). The same order of variation in the degree of crystallinity was also obtained from the intensity of the peak at 841 cm^−1^ (0.057 for PP, 0.038 for PP–CGHP, 0.104 for PP–CVBP, and 0.059 for PP–CGP). The results obtained by FTIR correlate very well with the results for the crystallinity index obtained by XRD in the case of PP, PP–CGP, and PP–CHGP composites. In the case of the PP–CVBP composite, the highest crystallinity value was obtained by FTIR, while the lowest value was obtained by XRD.

#### 3.2.3. Thermogravimetric Analysis

**Figure 7:**
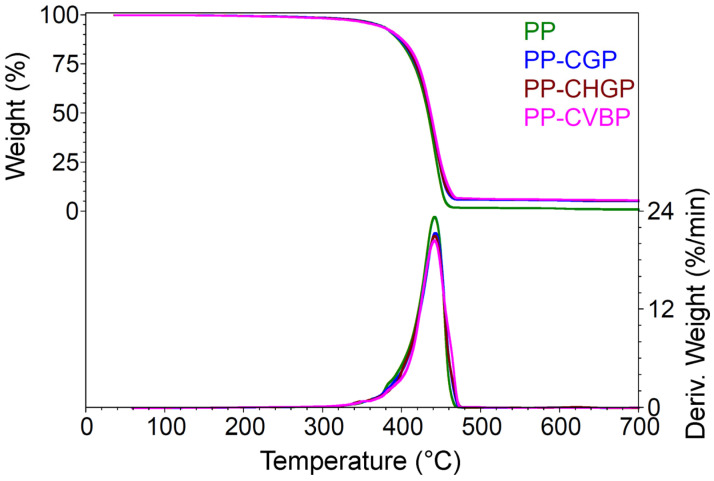
TGA curves of the PP, PP–CGP, PP–CHGP, and PP–CVBP composites.

TGA results (Figure 7) showed that the thermal stability of the composites was similar with that of the neat PP and the temperature at the maximum rate of decomposition differed by a maximum of 1 °C (Table 6).

From Table 6, it is also noticeable that the weight loss for the composites and neat PP, up to 230 °C, was small (0.5–0.6%), while the temperature at which the samples lost 5% of their weight varied only by 1–3 °C. The residue at 700 °C makes the difference between the samples. It can be seen that the PP–CHGP sample had the highest residue and the PP–CGP sample had the lowest. These results are a measure of the degree of interaction between the components and correlate with the TGA results obtained for the treated ashes.

#### 3.2.4. Differential Scanning Analysis

**Figure 8:**
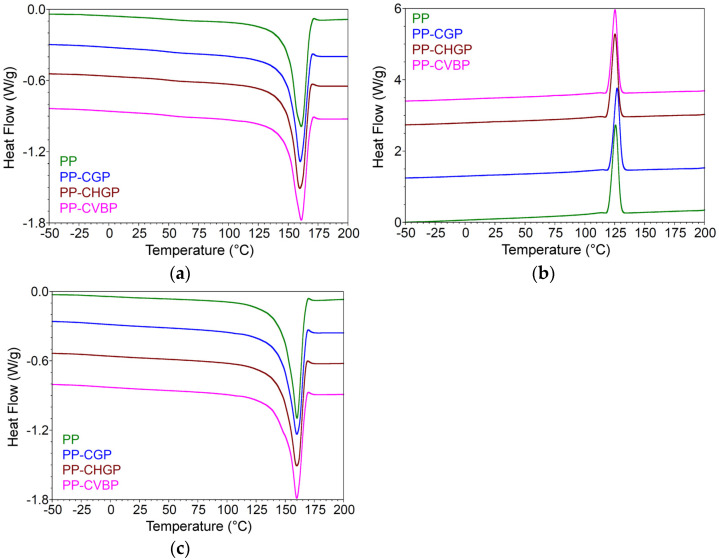
DSC thermograms of the PP, PP–CGP, PP–CHGP, and PP–CVBP composites, first heating (**a**) and cooling (**b**) cycle and second heating cycle (**c**).

The DSC thermograms (melting and crystallization curves from the first heating and cooling cycles and melting curves from the second heating cycle) of the PP and PP composites are shown in Figure 8. The main results, specific for the α crystalline phase of the PP (Tm, Tc, and Xc) are presented in Table 7. A small variation of Tm and Tc can be observed; in general a decrease of Tm by 0.9–1.3 °C on the first heating cycle and almost constant (insignificant variations of 0.1–0.2 °C) on the second heating cycle, in comparison with PP. The addition of ash slightly reduced Tc (by 0.5 °C in the case of PP–CVBP and PP–CHGP composites). The exceptions were the PP–CVBP composite, which maintained its Tm, and the PP–CGP composite, where Tc increased by 1.3 °C. The degree of crystallinity, Xc, is influenced by the degree of interaction of the components and varies in the order PP > PP–CGP ≥ PP–CHGP > PP–CVBP. A similar thermal behavior of polypropylene with more than 20 wt% fly ash was also reported by Nath et al. [18]. These results are in agreement with those obtained by XRD and represent further evidence of the existence of interactions between the treated ash, PP–MA, and the PP matrix.

#### 3.2.5. Mechanical and Dynamic Mechanical Analysis

**Figure 9:**
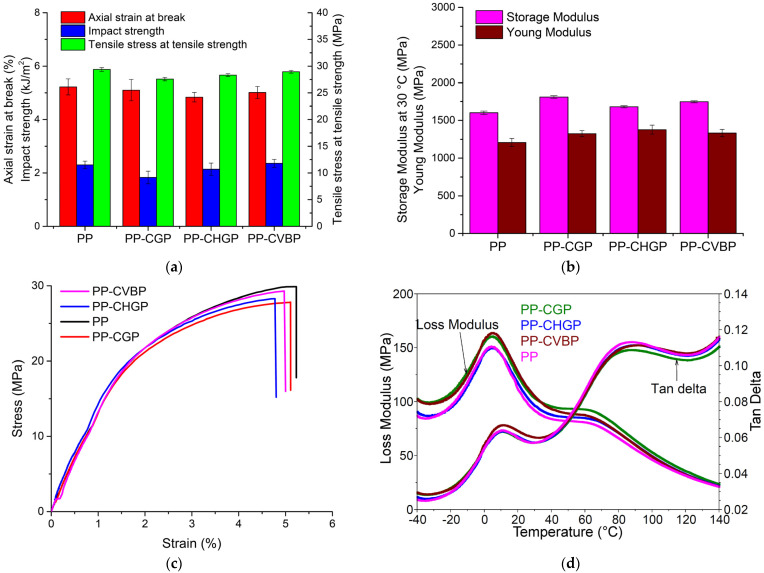
Mechanical and dynamic mechanical properties of PP, PP–CGP, PP–CHGP, and PP–CVBP composites. (**a**) Tensile and impact properties; (**b**) Young’s modulus and storage modulus; (**c**) stress vs. strain curves; (**d**) loss modulus and loss factors.

One of the most important aspects of composite manufacturing is monitoring the variation of properties between the composites and comparing them to a clean, neat sample of polymer. The degree of interaction, on the one hand, between the ash and the P agent (probably through the -OH groups) and, on the other hand, between the treated ash and the other components in the composite (probably through the ester groups with maleic anhydride in PP–MA and through propylene groups with the polymer matrix), is reflected in the value of the mechanical properties. Both axial strain and tensile strength values decrease by 2.5–7% and 1.5–6%, respectively, for the composites compared to recycled PP, as shown in Figure 9a. The impact strength decreases by 20% and 7% in the case of the PP–CGP and PP–CHGP composites, respectively, and is almost unchanged (a slight increase of 3%) in the case of the PP–CVBP composite. Instead, the modulus of elasticity of the composites increased by 10–14% (Figure 9b). Gummadi et al. [19] studied the flexural behavior of PP composites with ash and showed that by adding 10% ash with small particles (53–75 µm) and showed the flexural modulus and strength can be increased by 5% and 27%, respectively, but with a decrease in elongation at break by 18%. With larger particles (76–105 µm), the elongation at break decreased by half, and the flexural modulus decreased by 5%. The effect of ash on the tensile and impact properties of recycled PET was studied by Joseph et al. [21]. With 5% ash with particle sizes of 63–90 µm, a slight increase of 3–4% in tensile strength was obtained, but with decreasing in both elongation at break and impact strength by 45% and respectively 35%. With 15% fly ash treated with 1% aminosilane, a substantial improvement in the impact strength of 170% was obtained.

The obtained mechanical properties correlate very well with the degree of interaction between the components, the degree of crystallinity, and the composites’ thermal stability. The XRD and TGA results proved that, among the three composites, the PP–CHGP composite shows the smallest decrease in intensity of the peak corresponding to the α crystalline form of PP and the best thermal stability due to the good interaction between the components. This behavior is reflected in obtaining the highest value for stiffness (Young modulus), with the lowest tensile deformation and maintaining mechanical resistance (tensile strength) (Figure 9c). The PP–CVBP composite shows approximately the same stiffness and strength as the PP–CHGP composite (small differences of 2–3%) but the highest value for the impact strength (Figure 9a,b). The values obtained for the impact strength correlate with the XRD results (the impact strength increases in the order of maintaining the intensity of the peak corresponding to the β crystalline form of PP, namely PP–CVBP > PP–CHGP > PP–CGP).

The effect of treated ashes on the visco-elastic behavior of recycled PP was investigated by DMA. Thus, the stiffness and elastic behavior of the composites were evaluated by the storage modulus (E′), the viscous property, the loss modulus (E″), the impact resistance of the composites, the weight of the elastic and viscous phase, and the loss factor (tanδ) [33]. The storage modulus at 30 °C compared to the Young’s modulus is shown in Figure 9b, the loss modulus and loss factor of the PP composites as a function of temperature are shown in Figure 9d. The E′ of the PP composites increased by 9–13% compared to neat PP and was consistent with Young’s modulus (Figure 9b). The PP and PP composites showed similar loss modulus and loss factor curves. However, the samples can be differentiated based on the two relaxations observed in the loss modulus vs. temperature and loss factor vs. temperature curves for the PP and PP composites (Figure 9d). The first relaxation is related to the glass transition of the PP (Tg), and the second is related to lamellar slip and rotation in the crystalline phase of the PP [25]. The highest peak of the loss modulus curve, associated with Tg, is observed at about 5 °C. The PP composites showed higher Tg values by 1–1.3 °C compared to the PP, indicating a good interaction between the components. The highest Tg value was obtained for PP–CVBP and also the highest value for the loss modulus (Table 8), thus showing an improvement of the viscous properties due to a strong polymer matrix-ash adhesion. A study conducted by Sumita et al. [34] investigated the influence of particle size and volume fraction of ultrafine SiO_2_ on the dynamic mechanical properties of polypropylene and their study concluded that the broad transition which appears at around 60 °C is very likely to be related to the grain boundary of the PP composites.

A peak at around 10 °C can be observed on the tan δ vs. temperature plot that is associated with the glass transition temperature (Tg) of the PP. Tg has an insignificant variation (0.5 °C) when we compare the composites with the neat PP. A study performed by Joseph et al. [35] showed that at the same frequency (1 Hz) the values for tan δ firstly increase, reaching a maximum at Tg (which is associated with the β transition), then decrease and increase again reaching a maximum associated with the α transition. No significant change in both transitions of the PP was observed in the PP composites compared to neat PP (Table 8). However, a difference in the height of the tan δ peaks corresponding to the glass transition of the PP was observed. The higher peak value was observed for the PP–CVBP composite, indicating a more viscous characteristic and a good correlation with the highest impact strength value. The PP–CGP composite showed the lowest Tg value (from tan δ), indicating poor thermal stability [33], and the lowest tan δ value indicated low impact strength. This behavior is consistent with the TGA results (Table 6) and with the results of the mechanical impact tests (Figure 9a).

#### 3.2.6. Nanomechanical Analysis

**Figure 10:**
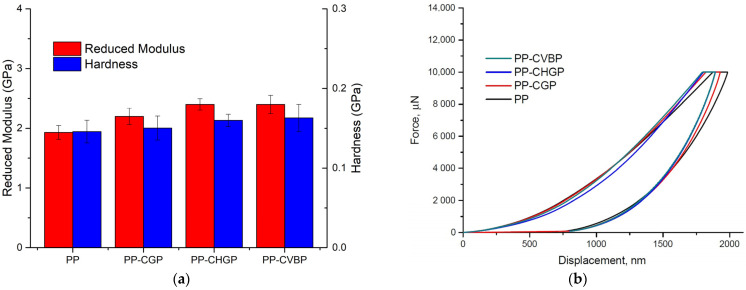
Nanomechanical properties of the PP, PP–CGP, PP–CHGP, and PP–CVBP composites; (**a**) reduced modulus and hardness; (**b**) force vs. displacement curves.

Nanomechanical analysis in Figure 10a showed an increase of reduced modulus ranging from 12 to 14% and an increase of hardness ranging from 3 to 10% for the composites with ash powder content compared to neat PP. These results are consistent with the values obtained for the Young’s modulus and storage modulus (Figure 9b).

The force-displacement curves in Figure 10b confirm that PP has a higher degree of elastic recovery compared to the composites, although the differences are not major between the samples. This elastic behavior correlates with what we already confirmed from previous mechanical and dynamic mechanical analyses regarding the elasticity of our composites. Several studies [36,37] performed on comparable material compositions have shown similar results where the reduced modulus and hardness increase with the addition of fillers compared to neat polymer.

**Figure 11:**
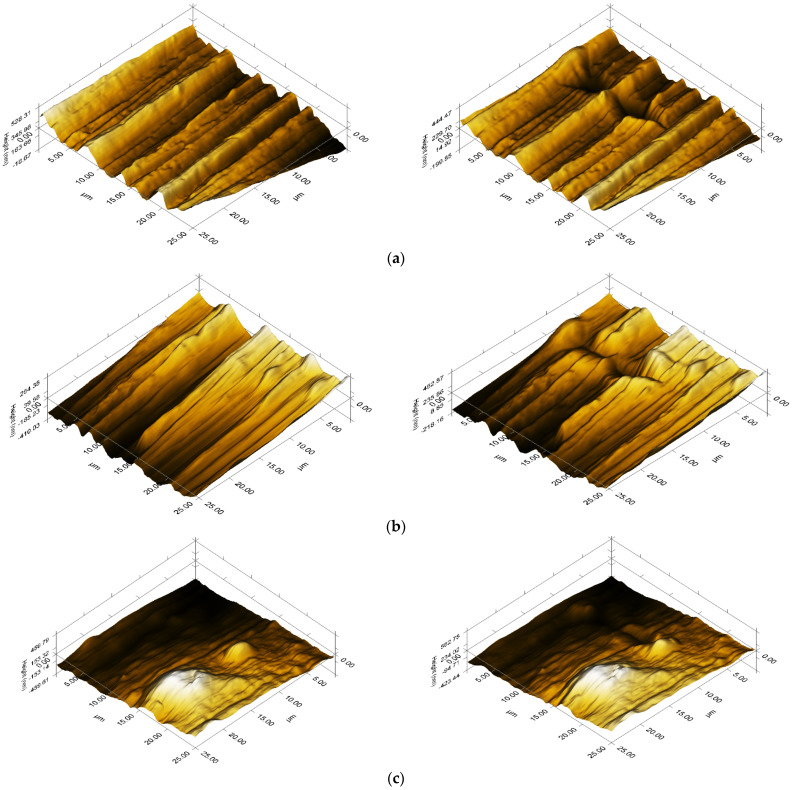

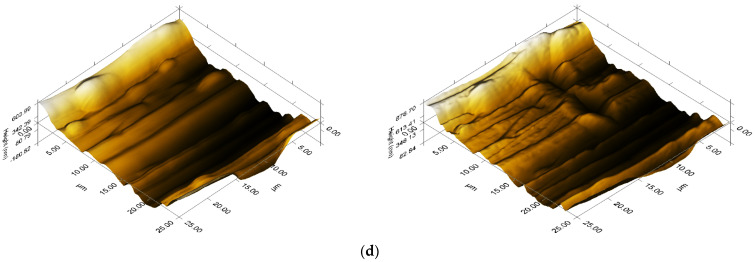
The 3D plot of the SPM image, before (**Left**) and after scratching (**Right**) for (**a**) PP; (**b**) PP–CGP; (**c**) PP–CHGP; (**d**) PP–CVBP.

Nanoscratching was performed to see changes in the surface topography under mechanical stress and to obtain more data regarding the roughness and coefficient of friction for the samples. Scanning probe microscopy (SPM) showed in Figure 11a–d that all composites have similar surface orientations in the form of surface structures that stretch as lines in the same direction.

This is due to the melt processing method when the granulated samples were injected under high temperature and pressure into a mold to form the standard tensile test specimens, which were later tested. Perpendicular to this orientation, the before and after images can be seen for each sample, where the Berkovich indenter tip has penetrated the surface and performed a nanoscratch according to the implemented load function. There are no signs of agglomeration along or at the beginning or end of the scratch, suggesting that the composites do not have brittle surfaces that would tear off under friction.

Furthermore, Table 9 shows that the roughness of each sample increases after the scratch since the nanomechanical test creates a further irregularity in the surface topography.

All three composites increased values of roughness before and after scratching but had similar values for the coefficient of friction, suggesting that they would have similar wear resistances.

#### 3.2.7. SEM and EDX Analysis

SEM analysis was performed on the fractured tensile specimens after they were sputter-coated with a thin layer of gold (5 nm).

**Figure 12:**
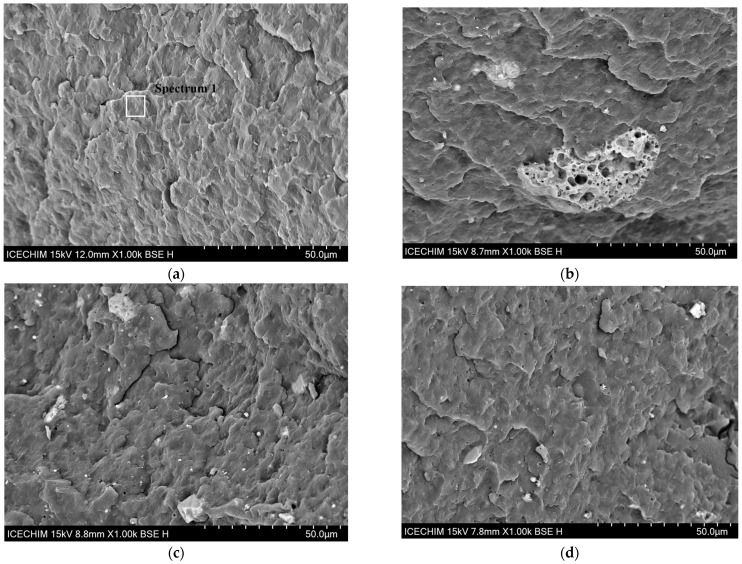
SEM surface morphology of (**a**) neat PP; (**b**) PP–CGP composite; (**c**) PP–CHGP composite; (**d**) PP–CVBP composite.

Figure 12a shows that the general structure of PP is clean and does not present major impurities or agglomerates.

Figure 12b–d, which represents PP–CGP, PP–CHGP and PP–CVBP, shows the presence of ash particles dispersed more or less uniformly in the PP matrix. A uniform dispersion of ash particles is observed in the case of PP-CGHP and PP–CVBP composites (Figure 12c,d). Particles with sizes between 1–10 microns are well anchored in the PP matrix, proving a strong interaction with it. If the composite is subjected to mechanical stress, the existence of these strong interactions will allow the efficient transfer of mechanical stress from the polymer matrix to the reinforcing agent. In the case of the PP–CGP composite, large and porous agglomerates of particles (15–40 microns) were observed, therefore, an uneven dispersion of ash particles due to poor polymer-ash matrix adhesion (Figure 12b). These images are in agreement with the results obtained for the mechanical properties of the composites.

## 4. Conclusions

In this work, recycled polypropylene (PP) from recovered face mask waste and silico-aluminous industrial waste were used to obtain composites with possible industrial applications. The addition to PP of 5 wt% from one of the three types of ash tested—two of the same origin (thermal plant ash—collected from the electro filter (CG) respectively collected from the landfill (CHG)) and the third was an ash from the process of obtaining basalt wool (CVB), contributed to obtaining composites with improved thermal and mechanical properties.

The interfacial adhesion between the polymer matrix and the ash was achieved by adding a compatibilizing agent, PP–MA, and by treating the ash surface with poly(propylene glycol adipate) (P). Different behaviors of the ashes were observed when treated with agent P. The strongest interaction with agent P was obtained for the ashes with the finest particles and the highest specific surface area (CHGP and CVBP). This good interfacial adhesion was further reflected in the uniform dispersion of the treated ash in the polymer matrix and, finally, in the improvement of the thermal stability, strength, and stiffness of the corresponding composites (PP–CHGP and PP–CVBP) compared to the PP–CGP composite.

With 6.5 wt% CHGP, the highest stiffness value was obtained (approx. 15% higher than PP), with a small decrease in impact strength (7%). A slightly improved impact strength value (3%) was obtained for the PP–CVBP composite and an increase in stiffness by 10%. In the case of the PP–CGP composite, a 10% increase in stiffness was obtained, but at the expense of tensile and impact strength, which decreased by 6% and 20%, respectively.

This study has shown that worn face masks can be used to obtain a viable polymer matrix for further use in industry. The addition of industrial aluminosilicate waste to recycled polypropylene from used face masks and homogenization by melt processing method results in increased stiffness and even impact strength without significantly reducing tensile strength. Further investigations are needed to valorize the studied materials and to find specific applications in an industry.

## 5. Patents

A patent application regarding the composition and processing method for composites obtained from face masks and ash powder was submitted to the State Office for Inventions and Trademarks.

## Figures and Tables

**Table 1:** Oxidic chemical composition and physical characteristics for CHG, CVB, and CG ash powder.

Characteristic	CHG	CVB	CG
**Oxidic Chemical Component, (%)**			
Loss on ignition (LOI)	7.85	12.35	3.08
SiO_2_	50.76	40.19	56.24
Al_2_O_3_	22.87	3.4	22.44
Fe_2_O_3_	6.49	6	8.03
CaO	4.49	1.5	5.65
MgO	2.40	7.8	0.08
SO_3_	0.08	1	1.33
Na_2_O	0.40	8.92	0.56
K_2_O	2.15	18.05	2.22
SiO_2_ reactive	40.25	39.74	48.11
**Physical Characteristics**			
Average diameter for 50% of particles, (d50), μm	12.72	6.7	23.6
Density, g/cm^3^	2.24	1.97	2.08
Specific surface area (Blaine value), cm^2^/g	8519	14,492	4650
R_009_, %	0.0	0.0	6.0

**Table 2:** XRD results for Quartz (SiO_2_), PDF card No. 01-083-0539.

Sample Name	2θ	d-Value (Å)	Height (cps)	FWHM (°)	Size (Å)	h k l Planes
CG	20.77	4.27	1542.94	0.60	133.79	1 0 0
	26.55	3.35	7756.41	0.35	232.16	1 0 1
	36.53	2.46	963.36	0.50	168.04	1 1 0
	50.16	1.82	1197.79	0.48	181.01	1 1 2
CGP	20.77	4.27	1541.54	0.35	229.14	1 0 0
	26.56	3.35	8263.79	0.35	231.82	1 0 1
	36.46	2.46	717.41	0.24	349.9	1 1 0
	50.08	1.82	790.14	0.40	217.4	1 1 2
CHG	20.77	4.27	1799.27	0.36	226.66	1 0 0
	26.57	3.35	9902.87	0.35	234.71	1 0 1
	36.52	2.46	529.48	0.30	277.53	1 1 0
	50.11	1.82	1067.70	0.40	221.47	1 1 2
CHGP	20.79	4.27	1915.11	0.33	246.67	1 0 0
	26.65	3.34	9497.51	0.31	260.64	1 0 1
	36.58	2.45	672	0.38	218.55	1 1 0
	50.11	1.82	900.90	0.41	216.54	1 1 2

**Table 3:** XRD results for Halite (NaCl), PDF card No. 00-005-0628.

Sample Name	2θ	d-Value (Å)	Height (cps)	FWHM (°)	Size (Å)	h k l Planes
CVB	27.32	3.26	1030.58	0.42	194.26	1 1 1
	31.67	2.82	11,722.75	0.32	259.69	2 0 0
	45.43	1.99	6298.4	0.32	272.23	2 2 0
	56.44	1.63	1824.69	0.32	280.6	2 2 2
CVBP	27.36	3.26	777.68	0.26	311.94	1 1 1
	31.71	2.82	9139.36	0.31	266.74	2 0 0
	45.42	2.00	4856.34	0.31	279.61	2 2 0
	56.43	1.63	1336.63	0.35	260.88	2 2 2

**Table 4:** TGA results of treated and untreated ashes.

Sample	RT-100 °C	RT-230 °C	Onset Point		Tmax	Residue
	Wt.	Wt.	Temp	Weight		700 °C
	%	%	°C	%	°C	%
CG	99.81	99.61	509.2	99.26	562.2	98.25
CGP	99.74	97.26	326.6	97.51	359.2	76.06
CHG	99.25	98.74	515.3	97.64	666.5	92.99
CHGP	99.4	97.04	320.2	96.26	353.4	73.23
CVB	99.17	97.39	512.3	98.55	612.3	94.44
CVBP	99.37	96.37	299.4	95.47	336.7	74.28
P	97.83	93.37	350.3	91.45	386.6	1.24

**Table 5:** XRD results for the α and β crystalline phases of the PP in the PP composites.

		αPP (110)	βPP (300)	αPP (040)	αPP (120)	CaCO_3_ (104)	K_β_
PP	2θ	14.043	16.043	16.821	18.491	29.4246	10.35%
	d (Å)	6.302	5.520	5.266	4.794	3.0331	
	Height (cps)	65,583	19,394	76,309	26,078	16,058.84	
	Size (Å)	126.5	180	156.5	120.3	221.76	
PP–CVBP	2θ	14.016	16.048	16.828	18.488	29.4110	9.64%
	d (Å)	6.313	5.518	5.264	4.795	3.0344	
	Height (cps)	39,992	10,827	45,682	15,803	5835.20	
	Size (Å)	128.9	173	152.9	117	240.8	
PP–CHGP	2θ	14.047	16.29	16.844	18.498	29.4270	4.79%
	d (Å)	6.299	5.438	5.259	4.7957	3.03283	
	Height (cps)	35,982	5800	59,088	20,134	7376.68	
	Size (Å)	130.8	120	163.8	122.1	218.64	
PP–CGP	2θ	14.026	16.2	16.827	18.486	29.4018	4.05%
	d (Å)	6.309	5.467	5.260	4.795	3.0354	
	Height (cps)	47,331	4956	51,750	18,166	6864.91	
	Size (Å)	128.8	137	158.1	117.8	192.99	

**Table 6:** TGA results of the PP, PP–CGP, PP–CHGP, and PP–CVBP composites.

Sample	RT-230 °C Wt. Loss %	230–500 °C		500–700 °C		Residue 700 °C		Temp for Wt. Loss 5% °C
		Wt. Loss %	Tmax °C	Wt. Loss %	Tmax °C	(N_2_) %	(Air) %	
PP	0.52	97.71	441.6	0.69	606.3	1.09	1.07	371.3
PP–CGP	0.46	93.63	442.6	0.63	617.0	5.28	5.28	370.0
PP–CHGP	0.58	92.97	442.0	0.87	621.3	5.57	5.57	368.7
PP–CVBP	0.64	92.92	441.5	0.77	590.9	5.38	5.37	368.0

**Table 7:** DSC results for PP, PP–CGP, PP–CHGP, and PP–CVBP composites.

Sample	1st Heating				Cooling				2nd Heating			
	Melting				Crystallization				Melting			
	Onset	Tmax	ΔHm	Xc	Onset	Tmax	ΔHc	Xc	Onset	Tmax	ΔHm	Xc
	°C	°C	J/(g)	(%)	°C	°C	J/(g)	(%)	°C	°C	J/(g)	(%)
PP	148.4	160.9	87.88	46.7	130.0	125.7	96.24	51.1	149.1	160.0	94.76	50.3
PP–CGP	149.7	160.0	82.23	43.7	131.1	127.0	91.32	48.5	147.4	159.9	88.54	47.0
PP–CHGP	149.6	159.6	79.57	42.2	129.4	125.2	91.51	48.6	148.0	159.8	88.78	47.1
PP–CVBP	147.6	160.9	81.47	43.3	129.4	125.2	90.73	48.2	149.8	159.9	87.98	46.7

**Table 8:** Loss modulus and loss factor values for the PP and PP composites.

Sample	Loss Modulus, E″				Loss Factor			
	Temp.	E″ Peak 1	Temp.	E″ Peak 2	Temp.	Tan Delta	Temp.	Tan Delta
	°C	MPa	°C	MPa	°C	Tan δ Peak 1	°C	Tan δ Peak 2
PP	4.61	150.0	59.56	80.37	11.15	0.06364	88.13	0.1127
PP–CGP	4.85	159.7	58.59	92.35	10.66	0.06298	87.4	0.1083
PP–CHGP	5.09	148.6	59.32	84.34	11.15	0.06319	90.31	0.111
PP–CVBP	5.3	163.1	59.16	87.07	11.15	0.06656	90.84	0.1109

**Table 9:** Roughness and coefficient of friction parameters of the PP, PP–CGP, PP–CHGP, and PP–CVBP composites.

Sample	Roughness (Rms, nm)		µ
	Before Scratch	After Scratch	
PP	81.41	87.04	0.28 ± 0.01
PP–CGP	116.68	121.55	0.28 ± 0.01
PP–CHGP	209.8	227.27	0.29 ± 0.01
PP–CVBP	158.41	161.92	0.28 ± 0.01

## Data Availability

The data presented in this study are available on request from the corresponding authors.
